# Supplementary material for: Gut microbiota confers host resistance to obesity by metabolizing dietary polyunsaturated fatty acids
Source: Nat Commun. 2019 Sep 5;10:4007. doi: 10.1038/s41467-019-11978-0 (PMC6728375; doi:10.1038/s41467-019-11978-0)
Supplement: Supplementary file 1 — Supplementary Information [file 41467_2019_11978_MOESM1_ESM.pdf]

SUPPLEMENTARY INFORMATION

Gut microbiota confers host resistance to obesity by metabolizing dietary polyunsaturated fatty acids

Miyamoto *et al*.

***Corresponding author; Ikuo Kimura
Email: ikimura@cc.tuat.ac.jp

This PDF file includes:

Supplementary Figures 1–9

Supplementary Table 1, 2

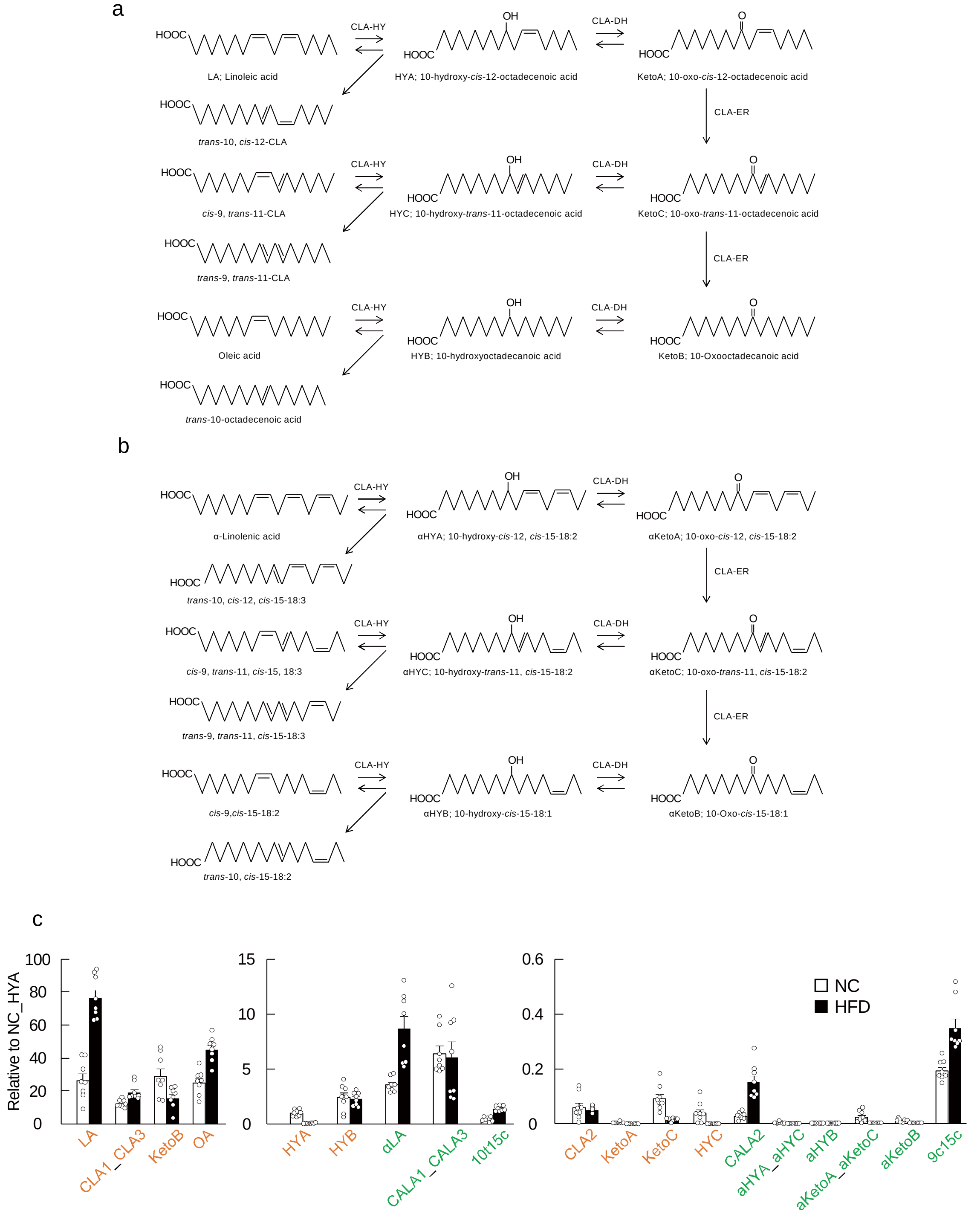

Supplementary Figure 1. Dietary PUFA-metabolic pathway mediated by gut microbiota. Gut microbial metabolites of (a) LA, and (b) αLA. (c) The relative abundance of gut microbial PUFA metabolites in cecal content (n = 8 per group). Results are presented as means ± SE. Open squares represent NC-fed mice and closed squares represent HFD-fed mice over 2 weeks. Source data are provided as a Source Data file.

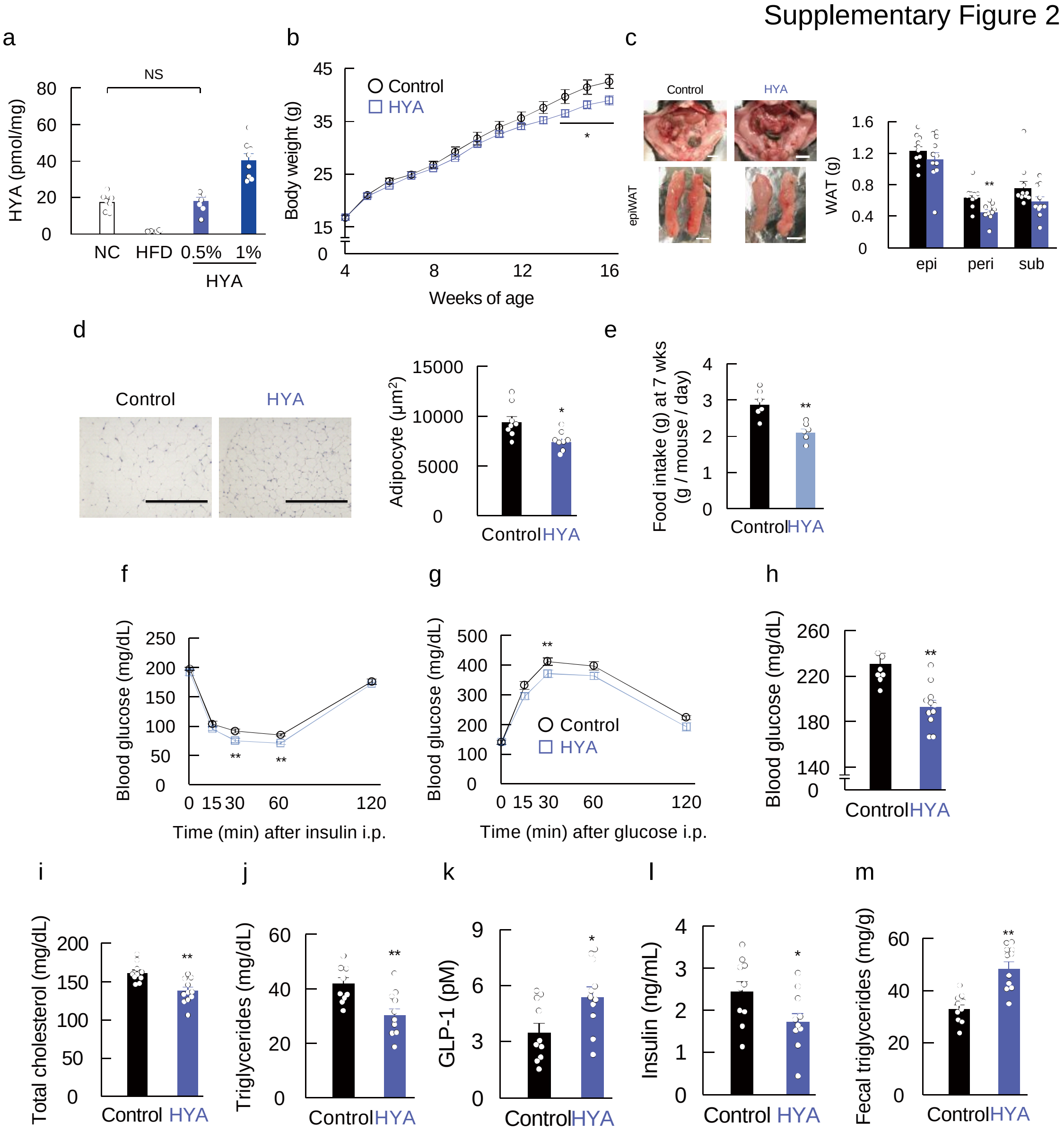

Supplementary Figure 2. Gut microbial PUFA metabolites improve host metabolic condition. (a) Quantification of HYA in cecal content (n = 8 per group). Changes in (b) body weight, representative macroscopic appearance, and (c) tissue weights were measured (n = 10 and 11 per group). Scale bar; 1 cm. epi, epididymal; peri, perirenal; sub, subcutaneous. (d) Hematoxylin-eosin (H&E)-stained WAT and mean area of adipocytes (n = 8 per group). Scale bar; 400 µm. (e) Daily food intake measured at 7 weeks of age (n = 6 per group). (f) ITT (n = 8 per group) and (g) GTT (n = 8 per group) were analyzed at 14 to 15 weeks of age. (h) Blood glucose, (i) total plasma cholesterol, (j) triglyceride, (k) GLP-1, and (l) insulin levels were measured at the end of the experimental period (n = 10 and 11 per group). (m) Fecal triglyceride levels were measured at 16 weeks of age (n = 10 and 11 per group). ***P* < 0.01; **P* < 0.05 vs. control (Student's *t* test). Results are presented as means ± SE. Source data are provided as a Source Data file.

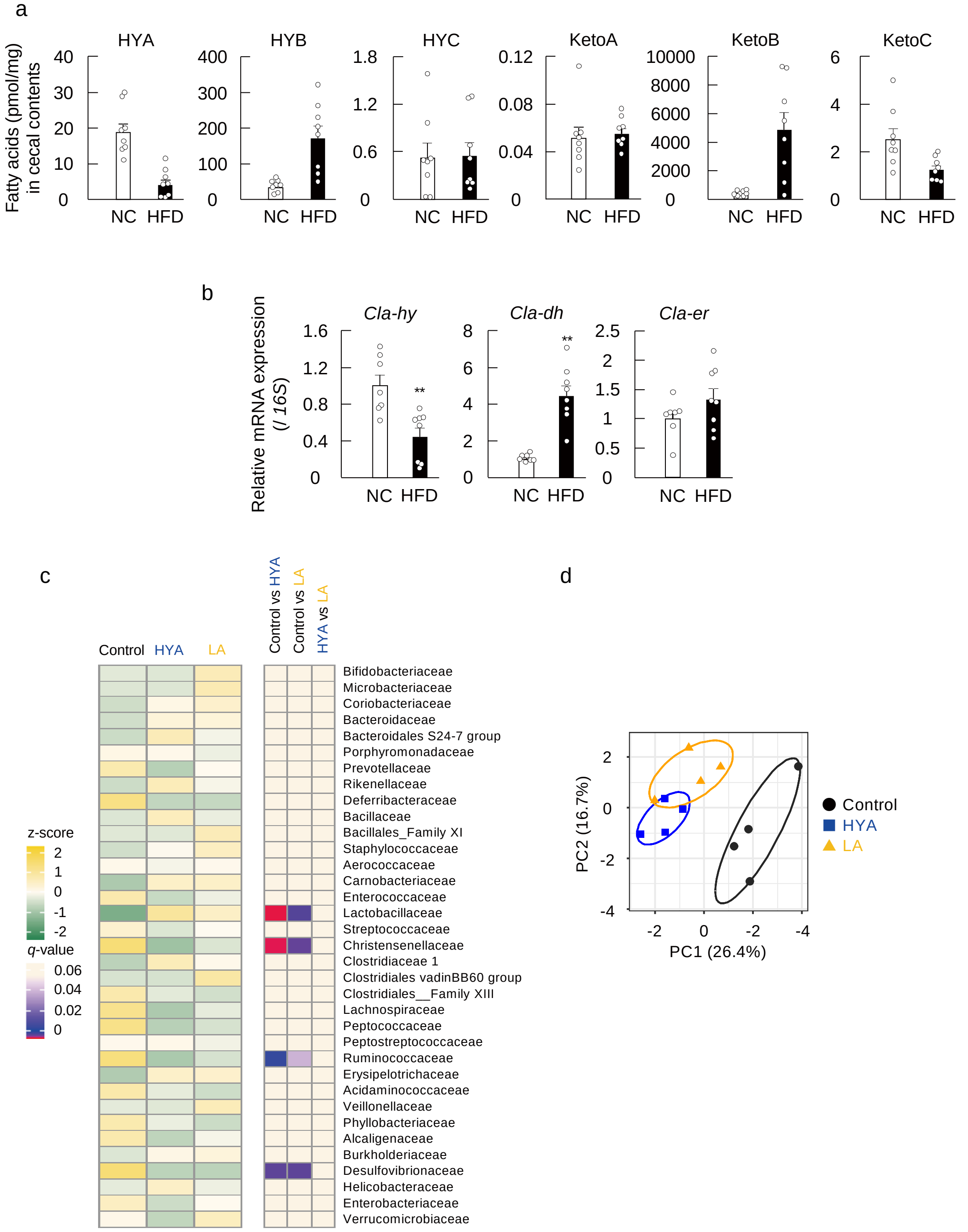

Supplementary Figure 3. Analysis of gut microbial metabolites and gut microbiota.

(a, b) Analysis of gut microbial metabolites of (a) LA and (b) the relative mRNA expression of metabolite-synthesizing enzymes (*Cla-hy*, *Cla-dh*, and *Cla-er*) (n = 7 and 8 per group). Open squares represent NC-fed mice, and closed squares represent HFD-fed mice over 12 weeks. (c, d) Gut microbial composition determined according to the abundance of bacterial domains at (c) the family level and (d) diversity (n = 4 samples per group). Source data are provided as a Source Data file.

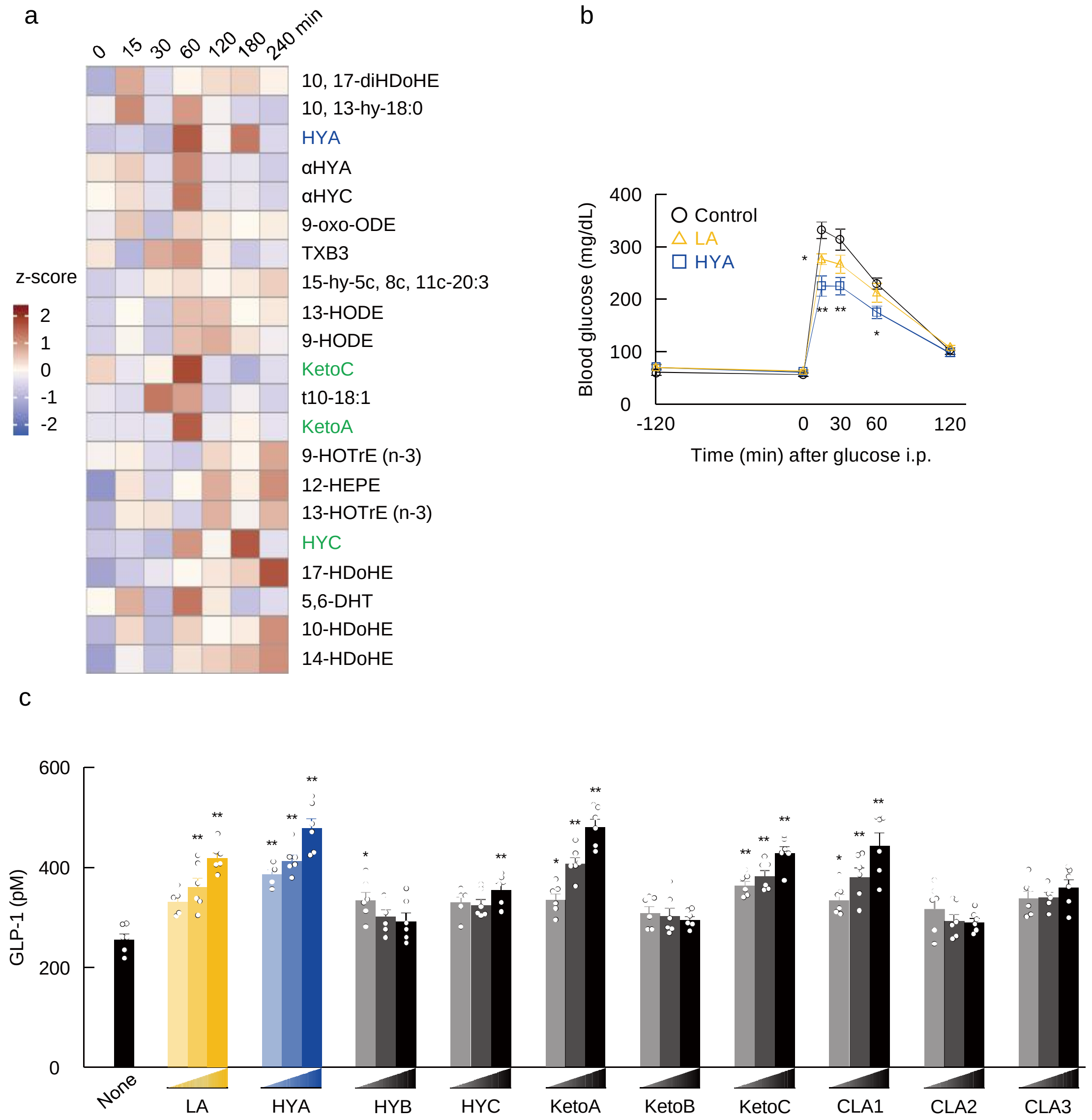

Supplementary Figure 4. Gut microbial metabolites regulate glucose homeostasis.

(a) Heat map of gut microbial PUFA metabolites in the ileum after oral administration of HYA (n = 3 tissues). (b) Individual FAs (HYA and LA; 1 g/kg) were administered by gavage, and IPGTT was analyzed (n = 8 animals). $^{**}P < 0.01$; $^{*}P < 0.05$ vs. control (Tukey–Kramer test). Results are presented as means ± SE. (c) GLUTag cells were treated with LA-derived gut microbial metabolites [in a dose-dependent manner (20, 100, and 200 μM)], and GLP-1 concentration was measured in culture medium (n = 6 independent cultures from two biological replicates). $^{**}P < 0.01$; $^{*}P < 0.05$ vs. None (Tukey–Kramer test). Results are presented as means ± SE. Source data are provided as a Source Data file.

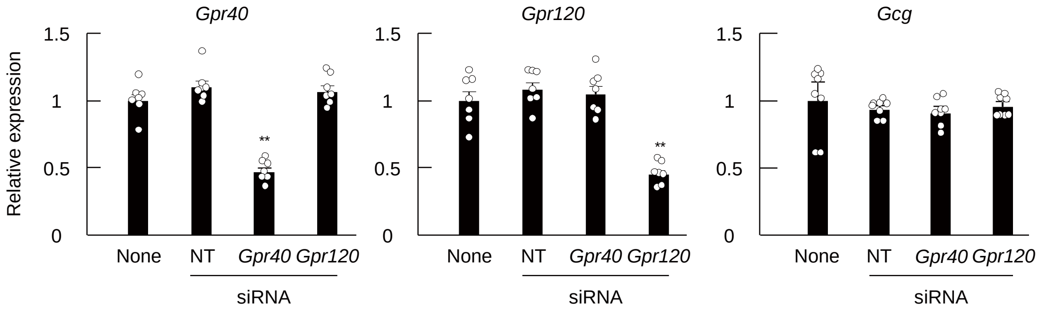

Supplementary Figure 5. The efficiency of siRNA knockdown STC-1 cells.

(a) mRNA expression of *Gpr40* (left), *Gpr120* (center), and *Gcg* (right) in individual siRNA-transfected STC-1 cells (n = 7 independent cultures for *Gpr40* and *Gpr120* from two biological replicates, n = 8 independent cultures for *Gcg* from two biological replicates). $^{**}P < 0.01$ vs. None (Tukey–Kramer test). Results are presented as means ± SE. NT, non-targeting siRNA. Source data are provided as a Source Data file.

Supplementary Figure 6

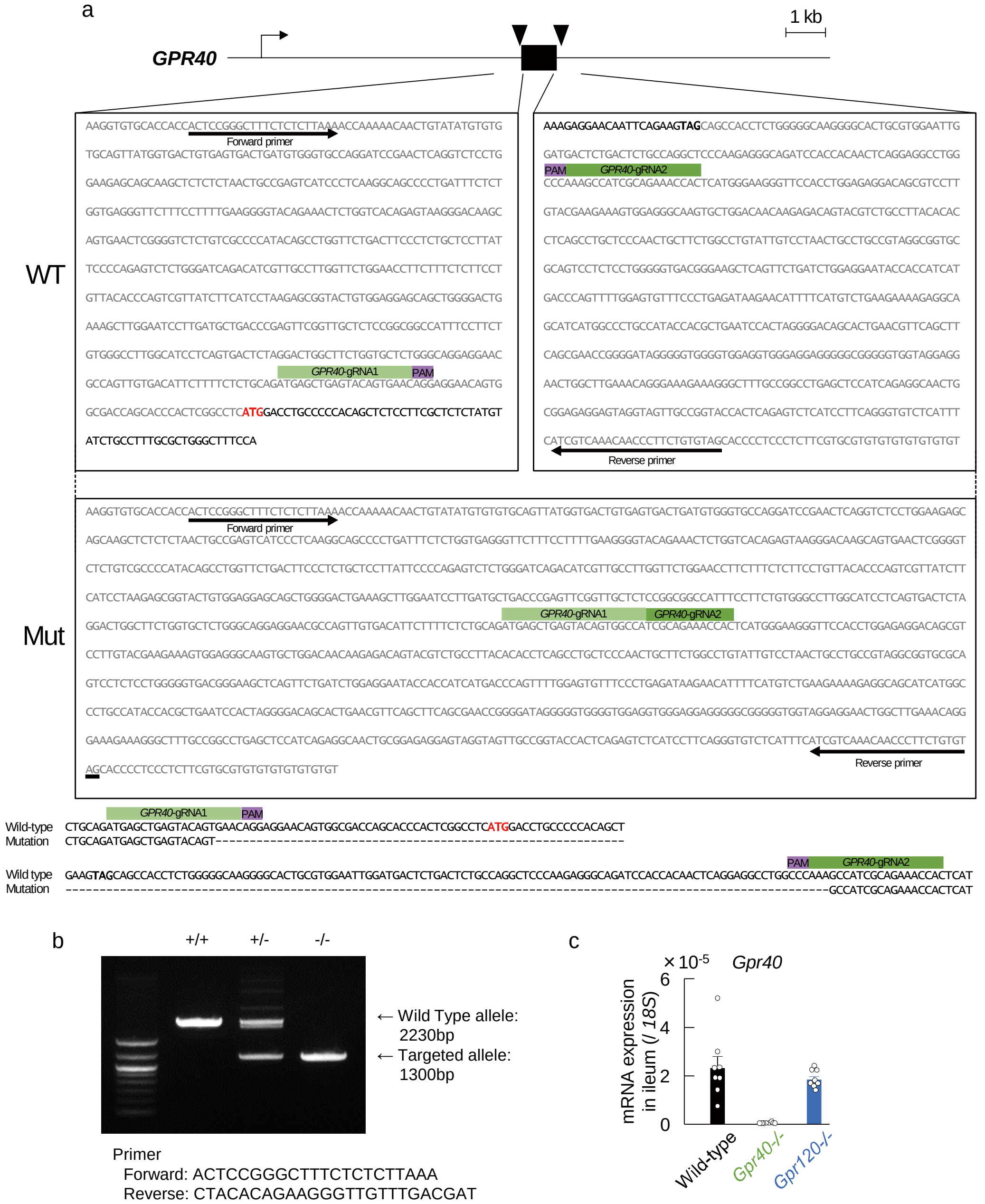

Supplementary Figure 6. *Gpr40* gene modification.

(a) Schematic representation of the genomic structures of *Gpr40* genes. *Gpr40*-deficient mice were generated using the CRISPR/Cas9 system in wild-type C57BL/6 zygotes. The coding exon region is shown in a black box. gRNA and PAM sequence are indicated in the green, and purple boxes, respectively. gRNA, guide RNA; PAM, protospacer-adjacent motif. (b) Mouse genotypes were determined by PCR using the indicated primers to detect wild-type and mutant *Gpr40* alleles. (c) mRNA expression of *Gpr40* in the ileum (n = 8 per group). Results are presented as means ± SE. Source data are provided as a Source Data file.

Supplementary Figure 7

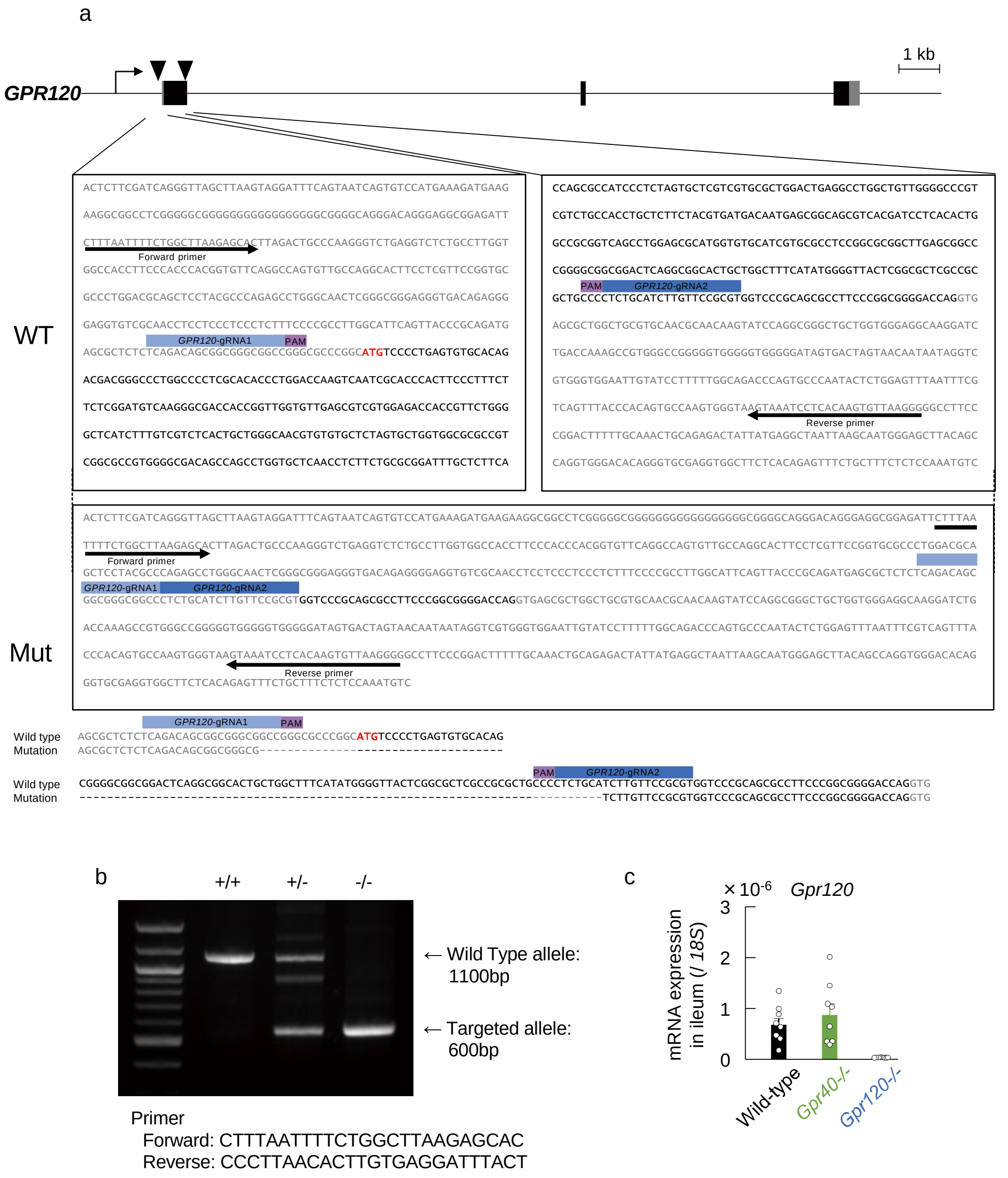

Supplementary Figure 7. *Gpr120* gene modification.

(a) Schematic representation of the genomic structure of *Gpr120* genes. *Gpr120*-deficient mice were generated using the CRISPR/Cas9 system in wild-type C57BL/6 zygotes. Coding and untranslated exon regions are shown as black and grey boxes and words, respectively. gRNA and PAM sequence are indicated in blue and purple boxes, respectively. gRNA, guide RNA; PAM, protospacer-adjacent motif. (b) Mouse genotypes were determined by PCR using the indicated primers to detect wild-type and mutant *Gpr120* alleles. (c) mRNA expression of *Gpr120* in the ileum (n = 8 per group). Results are presented as means ± SE. Source data are provided as a Source Data file.

Supplementary Figure 8

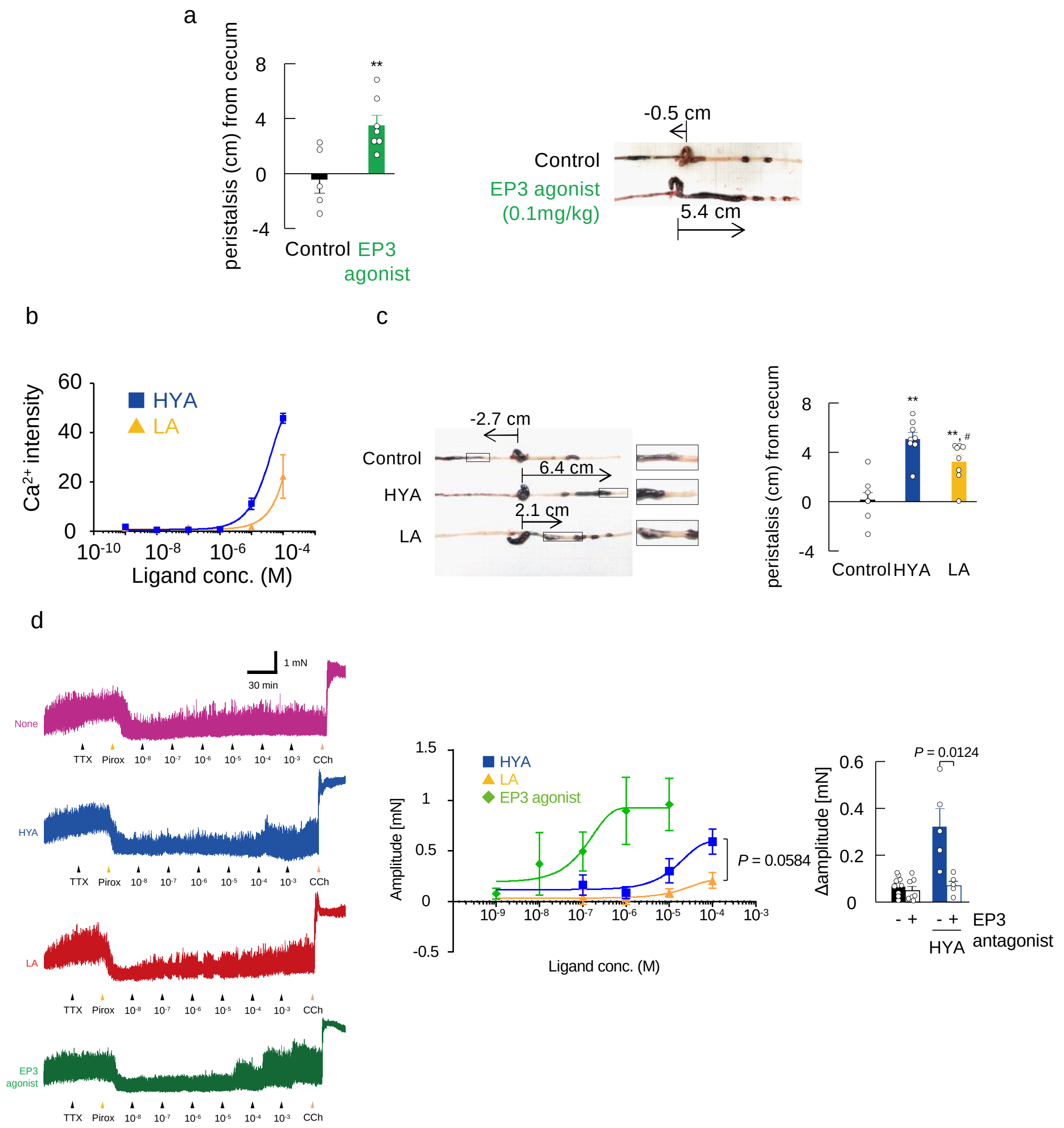

Supplementary Figure 8. HYA facilitates intestinal peristalsis via EP3 signaling.

(a) Intestinal peristalsis was assessed 2 h after administration of the EP3 agonist (sulprostone; 0.1 mg/kg) with charcoal by measuring the distance travelled by the charcoal (i.e., the base of the cecum or the start of the colon) (n = 5 and 7 animals per group). (b) Mobilization of $[Ca^{2+}]i$ induced by LA and HYA in EP3-experssiong Chem-1 cells. Data are presented as Ca^{2+} intensity (n = 3 independent experiments). (c) Intestinal peristalsis evaluated 2 h after FA administration (1 g/kg) with charcoal by measuring the distance travelled by the charcoal (i.e., the base of the cecum or the start of the colon) (n = 8 animals per group). (d) Representative traces of the isolated intestine in response to the cumulative addition of an HYA, LA, or EP3 agonist in the presence or absence of an EP3 antagonist (n = 10, 7, 5, and 5 samples per group). $^{**}P < 0.01$; $^{*}P < 0.05$ vs. None (Tukey–Kramer test). Results are presented as means ± SE. Source data are provided as a Source Data file.

Supplementary Figure 9

a

HYA-producing lactic acid bacteria.

HYA (+) bacteria	I.S.	LA (mg)	HYA (mg)	HYB (mg)
L. salivarius JCM1044	1	1.17±0.041	3.71±0.518	0.05±0.004
L. salivarius JCM1042	1	1.17±0.088	3.58±0.206	0.04±0.003
L. salivarius JCM1231	1	2.45±0.504	2.61±0.299	0.01±0.001
**HYA (-) bacteria**	**I.S.**	**LA (mg)**	**HYA (mg)**	**HYB (mg)**
L. johnsonii JCM1022	1	5.7±0.089	0.01±0.02	ND
L. johnsonii JCM8791	1	4.95±0.254	0.25±0.019	ND
L. acidophilus JCM1229	1	4.91±0.735	ND	ND

b

Colonization of Lactobacillus

	Lactobacillus genus (î 10^6 copies / ng DNA)
GF	ND
HYA (+)	5.9±0.827
HYA (-)	3.31±0.176

c

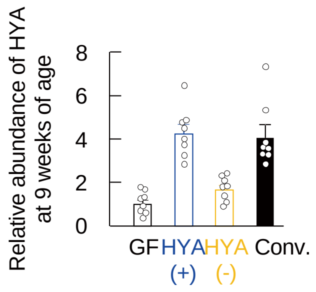

d

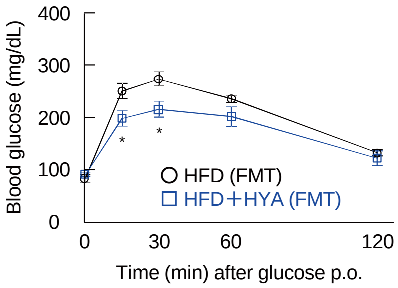

e

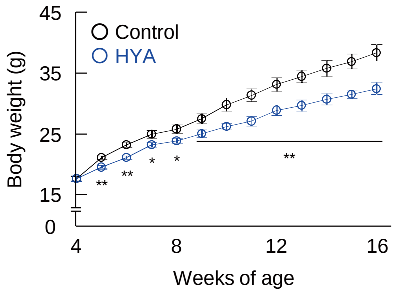

Supplementary Figure 9. HYA-producing lactic acid bacteria.

(a) Selection of HYA-producing *Lactobacillus* strains (n = 3 bacteria). (b) *Lactobacillus* colonization was detected by qPCR (n = 4 samples per group). (c) The relative abundance of HYA in feces was measured at 9 weeks of age (n = 8 samples per group). *P < 0.05 vs. GF mice (Tukey–Kramer test). (d) OGTT in FMT mice. Colonization was undertaken using fecal contents (suspended in sterile PBS) in conventional HFD-induced obese mice (n = 8 and 10 animals per group; 3 mice/pellet, 3 times/week). *P < 0.05 vs. HFD-fed gnotobiotic mice (Student's t test). (e) Changes in body weight under non-coprophagy conditions were measured (n = 9 animals per group). **P < 0.01; *P < 0.05 vs. control (Student's t test). Results are presented as means ± SE. Source data are provided as a Source Data file.

Screening of HYA-producing Lactobacillus

	I.S.	HYA (mg)	HYB (mg)
L. acidophilus JCM1028	1	0.005	ND
L. acidophilus JCM 1034	1	0.004±0.002	ND
L. acidophilus JCM 1132	1	0.023±0.004	ND
L. acidophilus JCM1229	1	0.004±0.002	ND
L. amylovorus JCM2125	1	0.041±0.002	ND
L. amylovorus JCM5811	1	0.018±0.001	0.002±0.002
L. antri JCM15950	1	0.055±0.004	ND
L. crispatus JCM8778	1	0.002±0.002	ND
L. gasseri JCM1025	1	0.023±0.005	0.002±0.002
L. gasseri JCM1130	1	0.035±0.003	ND
L. gasseri JCM1131	1	0.034±0.003	0.004±0.004
L. johnsonii JCM1022	1	0.005	ND
L. johnsonii JCM8791	1	0.011±0.001	ND
L. johnsonii JCM8794	1	0.014±0.001	ND
L. paracasei subsp. *paracasei* JCM1109	1	0.061±0.01	ND
L. paracasei subsp. *paracasei* JCM1133	1	0.055±0.005	ND
L. reuteri JCM1112	1	0.02±0.009	ND
L. salivarius JCM1040	1	0.048±0.013	0.002±0.002
L. salivarius JCM1042	1	0.389±0.157	0.005±0.005
L. salivarius JCM1044	1	0.088±0.027	0.006±0.006
L. salivarius JCM1045	1	0.146±0.022	ND
L. salivarius JCM1231	1	0.322±0.075	0.016±0.008

Supplementary Table 1. Selection of HYA-producing *Lactobacillus* strains.

After bacterial cultivation, HYA and HYB were quantified in culture medium (n = 3 culture supernatants per bacteria). Source data are provided as a Source Data file.

Composition of diets.

Product	HFD gm%	0.5% HYA gm%	1% HYA gm%	LA gm%	Normal chow gm%
protein	26	25.87	25.74	25.74	25.48
Carbohydrate	26	25.87	25.74	25.74	48.92
Fat (Soybean oil and/or Lard)	35	34.83	34.65	34.65	4.61
LA	0	0	0	1	0
HYA	0	0.5	1	0	0

Fatty acid ratio in total fat	gm%	gm%	gm%	gm%	gm%
Saturated	32	31.52	31.04	31.04	22.02
Monounsaturated	35.97	36.93	37.88	34.9	25.07
Polyunsaturated	32.04	31.55	31.08	34.06	52.91

Ingredient of soybean oil and/or Lard	gm%	gm%	gm%	gm%	gm%
C10, Capric acid	0.01	0.01	0.01	0.01	0
C12, Lauric acid	0.03	0.03	0.03	0.03	0
C14, Myristic acid	0.39	0.38	0.37	0.37	0.04
C15, pentadecylic acid	0.03	0.03	0.03	0.03	0
C16, Palmitic acid	6.87	6.73	6.59	6.59	0.75
C16:1, Palmitoleic acid	0.47	0.46	0.45	0.45	0.06
C17, Margaric acid	0.12	0.12	0.12	0.12	0.02
C18, Stearic acid	3.7	3.63	3.55	3.55	0.1
C18:1, Oleic acid	11.91	11.68	11.44	11.44	0.97
C18:2, Linoleic acid	10.06	9.86	9.66	9.66	2.05
C18:3, Linolenic acid	0.72	0.7	0.69	0.69	0.15
C20, Arachidic acid	0.06	0.05	0.05	0.05	0.02
C20:1	0.21	0.2	0.2	0.2	0.03
C20:2	0.28	0.27	0.26	0.26	0
C20:3	0.04	0.04	0.04	0.04	0
C20:4, Arachidonic acid	0.1	0.09	0.09	0.09	0
C22:5, Docosapentaenoic acid	0.03	0.03	0.03	0.03	0
C22:6, Docosahexaenoic acid	0	0	0	0	0.06
C24:0, Lignoceric acid	0	0	0	0	0.01
C24:1, Tetracosenoic acid	0	0	0	0	0.01
C18:2, Linoleic acid (free fatty acid)	0	0	0	1	0
C18:1 (OH), HYA (free fatty acid)	0	0.5	1	0	0

Supplementary Table 2. Diet compositions
