# Supplementary material for: Gut microbiota confers host resistance to obesity by metabolizing dietary polyunsaturated fatty acids
Source: Nat Commun. 2019 Sep 5;10:4007. doi: 10.1038/s41467-019-11978-0 (PMC6728375; doi:10.1038/s41467-019-11978-0)
Supplement: Supplementary file 3 — Reporting Summary [file 41467_2019_11978_MOESM3_ESM.pdf]

nature research

Corresponding author(s): Ikuo Kimura

Reporting Summary

Nature Research wishes to improve the reproducibility of the work that we publish. This form provides structure for consistency and transparency in reporting. For further information on Nature Research policies, see Authors & Referees and the Editorial Policy Checklist.

Statistical parameters

When statistical analyses are reported, confirm that the following items are present in the relevant location (e.g. figure legend, table legend, main text, or Methods section).

n/a	Confirmed	
☐	☒	The exact sample size (*n*) for each experimental group/condition, given as a discrete number and unit of measurement
☐	☒	An indication of whether measurements were taken from distinct samples or whether the same sample was measured repeatedly
☐	☒	The statistical test(s) used AND whether they are one- or two-sided<br>*Only common tests should be described solely by name; describe more complex techniques in the Methods section.*
☐	☒	A description of all covariates tested
☐	☒	A description of any assumptions or corrections, such as tests of normality and adjustment for multiple comparisons
☐	☒	A full description of the statistics including central tendency (e.g. means) or other basic estimates (e.g. regression coefficient) AND variation (e.g. standard deviation) or associated estimates of uncertainty (e.g. confidence intervals)
☒	☐	For null hypothesis testing, the test statistic (e.g. *F*, *t*, *r*) with confidence intervals, effect sizes, degrees of freedom and *P* value noted<br>*Give P values as exact values whenever suitable.*
☒	☐	For Bayesian analysis, information on the choice of priors and Markov chain Monte Carlo settings
☒	☐	For hierarchical and complex designs, identification of the appropriate level for tests and full reporting of outcomes
☒	☐	Estimates of effect sizes (e.g. Cohen's *d*, Pearson's *r*), indicating how they were calculated
☐	☒	Clearly defined error bars<br>*State explicitly what error bars represent (e.g. SD, SE, CI)*

Our web collection on statistics for biologists may be useful.

Software and code

Policy information about availability of computer code

Data collection	Microscopy: EVOS FLc (Thermo Fisher Scientific), BZ-X700 (KEYENCE).<br>QPCR: StepOne (Applied Biosystems).<br>Illumina sequencing: Illumina Miseq. Sequences were aligned to the Silva reference database using QIIME.<br>Metabolites analysis: QTRAP 5500 (AB SCIEX), Xevo TQD (waters)
Data analysis	Bioinformatic and statistical analysis were used with R package (version 3.4.3).<br>Image J (NIH) was used to measure adipocyte diameter using stained sections of adipose tissue.

For manuscripts utilizing custom algorithms or software that are central to the research but not yet described in published literature, software must be made available to editors/reviewers upon request. We strongly encourage code deposition in a community repository (e.g. GitHub). See the Nature Research guidelines for submitting code & software for further information.

Data

Policy information about availability of data

All manuscripts must include a data availability statement. This statement should provide the following information, where applicable:

- Accession codes, unique identifiers, or web links for publicly available datasets
- A list of figures that have associated raw data
- A description of any restrictions on data availability

Associated raw data is indicated in the appropriate figure legends and a data availability statement is provided in the materials and methods section.
Fastq files of the Illumina short read sequences used in the commensal bacteria composition analysis will be made available on DNA Data Bank of Japan (DDBJ) under the following BioProject accession number: DRA008263 (Figure 1), and DRA008264 (Supplemental Figure 3).
The raw data of lipid metabolome have been deposited into the National Institutes of Health for Metabolomics Workbench under the DataTrack ID1695 (Figure 4) and DataTrack ID1702 (Supplemental Figure 4).

Field-specific reporting

Please select the best fit for your research. If you are not sure, read the appropriate sections before making your selection.

☒ Life sciences ☐ Behavioural & social sciences ☐ Ecological, evolutionary & environmental sciences

For a reference copy of the document with all sections, see nature.com/authors/policies/ReportingSummary-flat.pdf

Life sciences study design

All studies must disclose on these points even when the disclosure is negative.

Sample size	In our experience, the sample sizes chosen are sufficient to obtain reliable results. These sample sizes are in lie with the standard practice for data published in this field. Samples are always pooled across multiple independent biological replicates. Statistical analysis was applied after data collection. Exact sample size number for each experiment is reported in the figure legends.
Data exclusions	None
Replication	For each experiment, the findings were reliably reproduced and reported in the figure legends and materials and methods.
Randomization	Data collection and analysis was carried out on randomly selected samples.
Blinding	Investigators were not formally blinded to group allocation during data collection or analysis. However, more than two independent investigators scored the results of an un-biased experiments that did not have an a priori hypothesis.

Reporting for specific materials, systems and methods

Materials & experimental systems

n/a	Involved in the study	
☐	☒	Unique biological materials
☐	☒	Antibodies
☐	☒	Eukaryotic cell lines
☒	☐	Palaeontology
☐	☒	Animals and other organisms
☒	☐	Human research participants

Methods

n/a	Involved in the study	
☒	☐	ChIP-seq
☒	☐	Flow cytometry
☒	☐	MRI-based neuroimaging

Unique biological materials

Policy information about availability of materials

Obtaining unique materials	All unique materials are readily available from authors or commercial sources as listed in the materials and methods.

Antibodies

Antibodies used	F4/80 antibody (Abcam, ab6640, CI:A3-1)<br>Caveolin-1 antibody (BD, 610406, 2297)
Validation	F4/80 antibody (https://www.abcam.co.jp/f480-antibody-cia3-1-ab6640.html)<br>Caveolin-1 antibody (http://www.bdbiosciences.com/eu/reagents/research/antibodies-buffers/cell-biology-reagents/cell-biology-antibodies/purified-mouse-anti-caveolin-1-2297caveolin-1/p/610406)

Eukaryotic cell lines

Policy information about cell lines

Cell line source(s)	STC-1 cells (ATCC), GLUTag cells (gifted by Drucker DJ), and Flp-In™ 293 T-REx cells (Invitrogen)
Authentication	This STC-1 cell lines have been authenticated by ATCC.<br>This Flp-In™ 293 T-REx cell lines have been authenticated by Invitrogen.
Mycoplasma contamination	Cell line cultures were free of mycoplasma contamination.
Commonly misidentified lines (See ICLAC register)	No commonly misidentified cell lines were used.

Animals and other organisms

Policy information about studies involving animals; ARRIVE guidelines recommended for reporting animal research

Laboratory animals	C57BL/6 (wild-type, Gpr40-/-, and Gpr120-/-) male mice.
Wild animals	Wild animals were not used.
Field-collected samples	Field-collected samples were not used.
